# The complete mitochondrial genome of a microalgae *Chlamydomonas moewusii* strain XJCH-01 from Tarim Basin of Xinjiang, China

**DOI:** 10.1080/23802359.2020.1731371

**Published:** 2020-02-28

**Authors:** Yan-Bin Li, Yan-Zhen Zhang, Ying Jin, Hong Zeng, Jia-Yu Duan, Ling-Xiao Liu, Yun-Guo Liu, Zhi-Hai Sui

**Affiliations:** a Xinjiang Production & Construction Corps Key Laboratory of Protection and Utilization of Biological Resources in Tarim Basin, Tarim University, Alaer, China;; b College of Life Sciences and Technology, Xinjiang University, Urumqi, China;; c College of Life Sciences, Linyi University, Linyi, China;; d Qingdao Customs District P.R.China, Qingdao, China;; e Linyi Academy of Agricultural Sciences, Linyi, China

**Keywords:** Microalgae, *Chlamydomonas moewusii*, Mitochondrial genome

## Abstract

*Chlamydomonas moewusii* is a microalga isolated from the Tarim Basin of Xinjiang, China. The complete mitochondrial genome sequence of *C. moewusii* strain XJCH-01 was determined in this study (Accession number MT015649). The mitogenome (22,887 bp, 34.58% G + C) consists of 7 protein-coding genes (PCG), discontinuous large and small subunit ribosomal RNA (rRNA), and 4 transfer RNA (tRNA) genes. The complete mitochondrial genome sequence of the *C. moewusii* strain XJCH-01 enriches data resources for further study in genetic and functional evolution.

The single-celled *Chlamydomonas* was similar to the ancestors of land plants, which is considered an interesting model organism for studying genetic and functional evolution (Merchant et al. 2007). *Chlamydomonas moewusii* is a genus of the family *Chlamydomonadaceae* in the order *Chlamydomonadales* of *Chlorophyceae* consisting of unicellular flagellates, found in stagnant water and on damp soil containing organic matter, as a hydrogen-producing ecologically important group in the environment (Harris 2001). Here, the complete mitochondrial genome of *C. moewusii* was sequenced and characterized in detail. The strain of *C. moewusii*, named as XJCH-01, was isolated from Tarim Basin (39°95′N 84°26′E), Xinjiang Uygur Autonomous region, China in August, 2019. It was stored in the College of Life Sciences, Linyi University, Linyi, China. Genomic DNA was extracted from cultured *C. moewusii* according to Liu et al. (2012, 2014). The complete mitochondrial genome of *C. moewusii* strain XJCH-01 was sequenced using a shotgun approach and assembly. Subsequently, sequence data were analyzed according to Denovan-Wright et al. (1998). Clustal W was used for multiple sequence alignments (Thompson et al. 1994) and nucleotide sequence similarity searches were performed at the National Center for Biotechnology Information using the BLAST online service (Altschul et al. 1990).

The complete mitochondrial genome *C. moewusii* strain XJCH-01 (Accession number MT015649) was 22,887 bp in length with a G + C content of 34.58%. The *C. moewusii* mtDNA encodes seven respiratory chain proteins (*cob*: apocytochrome b, *cox1*: subunit 1 of cytochrome oxidase, subunits *nad1*, *nad2*, *nad4*, *nad5*, and *nad6*: 1, 2, 4, 5, and 6 of the NADH dehydrogenase complex), four tRNAs and discontinuous LSU and SSU rRNAs. However, there are two large direct repeat regions that do not appear to encode any functional mitochondrial molecule. The arrangement and composition of the mitochondrial genome are similar to the known microalgae genomes (Wolff et al. 1994; Denovan-Wright et al. 1998; Smith et al., 2011; Servin-Garciduenas and Martinez-Romero 2012; Tourasse et al. 2015; Hu et al. 2019; Huang et al. 2019). However, it showed a big difference from the vertebrates (Liu et al. 2016; Li, Liu, Sui, et al. 2019; Li, Kiu, Zhang, 2019) and the Arthropoda (Lavrov et al. 2000; Masta and Boore 2004; Choi et al. 2007). A phylogenetic tree was constructed based on the comparison of the complete mitochondrial genome sequences with other *Chlorophyceae* species using neighbour-joining method to determine the phylogenetic position of *C. moewusii* strain XJCH-01 (Figure 1). All 7 protein-coding genes identified in the *C. moewusii* mitochondrial genome, started with the typical initiation codon ATG. No incomplete termination codons were found, all PCGs had the complete termination codons TAA, whereas *cob* terminate with TAG. Among them, three (*cob*, *cox1*, and *nad1*) each contain one group I intron and one (*nad5*) contains two group I introns. In addition, seven ORFs (i1-orf, i2-orf, i3-orf, i4-orf, i5-orf, i6-orf, i7-orf) were identified within the nine introns interrupting the coding regions of *C. moewusii* mtDNA. There is an additional gene for *tRNA-met* in *C. moewusii* mtDNA. Two of the tRNA genes, *tRNA-Trp* and *tRNA-Gln*, are clustered and separated by only 7 bp, whereas *tRNA-met-1* and *tRNA-met-2* were located between the two LSU regions, and separated by a large direct repeat region. The mitochondrial SSU and LSU rRNAs are encoded by three (*rns-a* ∼ *rns-c*) and six (*rnl-a* ∼ *rnl-f*) rRNA gene pieces, respectively, which were dispersed throughout the genome and interspersed with each other and with protein-coding and tRNA genes. All the mitogenome genes were encoded on the same strand of the mtDNA molecule. The complete mitochondrial genome sequence obtained in our study would facilitate further investigations of phylogenetic relationships, genomic evolution and function within *Chlorophyceae*.

**Figure 1.**
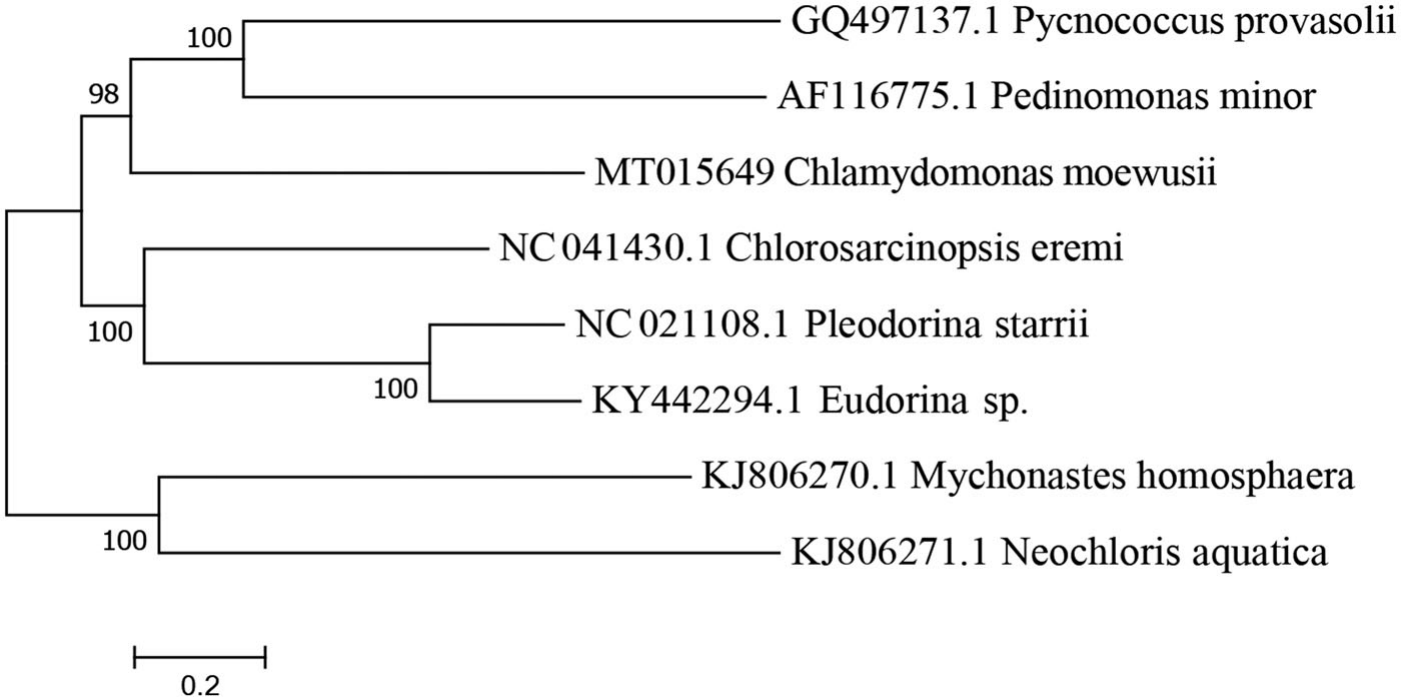
A phylogenetic tree constructed based on the comparison of complete mitochondrial genome sequences with of the other seven *Chlorophyceae* species. The seven *Chlorophyceae* species are *Pycnococcus provasolii*, *Pedinomonas minor*, *Chlorosarcinopsis eremi*, *Pleodorina starrii*, *Iudorina* sp., *Mychonastges homosphaera*, and *Neochloris aquatica*. Genbank accession numbers for all sequences are listed in the figure. The numbers at the nodes are bootstrap percent probability values based on 1000 replications.
